# Tumor immunology and cancer immunotherapy: summary of the 2014 SITC primer

**DOI:** 10.1186/s40425-015-0072-2

**Published:** 2015-06-16

**Authors:** David B. Page, Ariel Bulua Bourla, Anthony Daniyan, Jarushka Naidoo, Eric Smith, Melody Smith, Claire Friedman, Danny N. Khalil, Samuel Funt, Alexander N. Shoushtari, Willem W. Overwijk, Padmanee Sharma, Margaret K. Callahan

**Affiliations:** Memorial Sloan Kettering Cancer Center, 300 E 66th Street, BAIC 813, NY 10065 New York, USA; MD Anderson Cancer Center, Houston, TX USA

**Keywords:** CTLA4, PD-1, PD-L1, Melanoma, Prostate cancer, Kidney cancer, Bladder cancer, Lung cancer, Vaccine, Adoptive therapy, Nivolumab, Ipilimumab, Pembrolizumab, SITC, Primer

## Abstract

The pioneers of tumor immunology and cancer immunotherapy, including the late William B. Coley and Lloyd J. Old, have championed the potential for immunotherapy for over a century. Finally, advances in our understanding of the fundamentals of tumor immunology are translating into clinical success, with recent US Food and Drug Administration approval of several immunotherapies that improve clinical outcomes across prostate cancer, metastatic melanoma, non-small cell lung cancer and lymphocytic leukemia. In tandem with these clinical successes, new technologies such as high-throughput DNA/RNA sequencing, genetic engineering, and streamlined ex vivo cell culturing have paved the way for the next generation of immunotherapies and provided new tools for investigating potential biomarkers of response to existing therapies. During the November 2014 Annual Meeting of the Society of the Immunotherapy of Cancer, leaders in tumor immunology and cancer immunotherapy convened at the second annual SITC Primer to review both current knowledge and future directions in the field. Here, we will review the key discussions across a variety of topics, including innate immunity, adaptive immunity, dendritic cells, adoptive T cell therapy, anti-tumor antibodies, cancer vaccines, immune checkpoint blockade, challenges to immunotherapy, monitoring immune responses, and immunotherapy clinical trial design.

## Introduction

Clinical immunotherapy is undergoing a renaissance, with recent rapid-fire US Food and Drug Association (FDA) approval of numerous immunotherapies across several tumor types, including ipilimumab, pembrolizumab and nivolumab for metastatic melanoma, nivolumab for non-small cell squamous cell lung cancer, sipuleucel-T for prostate cancer, and blinatumomab for acute lymphocytic leukemia (ALL). In parallel, advances in research tools for basic and translational investigation have afforded an enhanced understanding of the fundamentals of tumor immunology, laying the foundation for subsequent development of the next generation of immunotherapeutic approaches. These approaches include therapeutic antibodies against novel targets, adoptive T-cell therapies, tumor vaccines, bispecific antibody constructs, and combination therapies. In November 2014, the Society for Immunotherapy of Cancer (SITC) held the second annual Primer on Tumor Immunology and Cancer Immunotherapy, organized by Drs. Willem W. Overwijk and Padmanee Sharma. In this review, we will summarize the key topics presented by the faculty, focusing upon recent advances in the field of immunotherapy. This year’s faculty included Dr. Vincenzo Bronte, MD (innate immunity), Dr. Lisa H. Butterfield, PhD (dendritic cells), Dr. Jonathan Powell, MD/PhD (adaptive immunity), Dr. Carl H. June, MD (adoptive T-cell therapy), Dr. Sattva S. Neelapu, MD (anti-tumor antibodies), Dr. Margaret K. Callahan, MD/PhD (obstacles to driving an immune response), Dr. Willem W. Overwijk, PhD (cancer vaccines), Dr. James P. Allison, PhD (targeting immune checkpoints in cancer therapy), Dr. Sasha Gnjatic, PhD (immune monitoring), and Dr. Padmanee Sharma, MD/PhD (pre-surgical clinical trial design).

### Innate immunity

The innate immune system is evolutionarily conserved across both vertebrates and non-vertebrates, and provides a rapid, but non-specific, protective immune response to invading pathogens, The innate immune system responds within minutes and does not generate memory, but notably can stimulate and shape long-lasting antigen-specific immunity through a variety of mechanisms. Principal components of innate immunity include epithelial barriers (skin and mucosal membranes), pattern recognition receptors (PRRs), effector cells (monocytes/macrophages, natural killer (NK) cells, dendritic cells (DCs), mast cells, neutrophils, innate lymphoid cells, and eosinophils among others) and humoral components (complement proteins and collectins).

The innate immune response can be initiated through activation of PRRs such as Toll-like receptors (TLR). These receptors can be extracellular, cytosolic, or endosomal. Pathogen associated molecular patterns (PAMP) are molecules shared by groups of pathogens (i.e. gram positive and gram negative bacteria), are not present in mammalian cells, and are capable of binding PRRs. These include bacterial cell wall structures such as lipopolysaccharide, as well as nucleic acids such as double-stranded RNA. Similarly, damage associated molecular patterns (DAMPs) such as HMGB1, DNA, RNA and S100 molecules can activate PRRs. Activation of PRRs leads to inflammatory cytokine release, which ultimately contributes to maturation of dendritic cells and presentation of antigens to the adaptive immune system. In tumors, anti-neoplastic therapies may promote tumor apoptosis, inflammation, and presentation of tumor-associated antigens with activation of anti-tumor T cell immunity. In contrast, persistent hypoxia in growing tumors leads to necrosis and chronic release of HMGB1, promoting angiogenesis and tumor growth through the recruitment of suppressive tumor-associated macrophages and endothelial precursor cells [1].

The innate immune response is driven by a number of cell types and mediated by cytokines including tumor necrosis factor (TNF), interleukin-1 (IL-1), IL-12 and interferon gamma (IFN-γ), among others. Macrophages have been classically implicated in microbicidal actions including phagocytosis and bacterial/fungal killing, as well as antigen presentation to T cells. However, macrophages may have positive or negative effects in the tumor microenvironment. In this regard, the distinction between classically activated M1 macrophages and alternatively activated M2 macrophages is an important one. Whereas M1 macrophages are responsible for phagocytosis and tumouricidal activity, M2 macrophages generate anti-inflammatory cytokines and play a role in wound repair and fibrosis, and may promote tumor survival, for example by promoting tumor vascularization and immune suppression [2,3]. NK cells also play an important role in innate immunity, as they are capable of killing injured cells, infected cells, and phagocytosed microbes. NK cells are activated by receptors which recognize stress-induced molecules on the cell surface, both in the setting of infection or neoplasm. NK cells also express inhibitory receptors which bind class I major histocompatibility complex (MHCI), and exhibit cytotoxic activity against tumors that aberrantly downregulate MHCI. Lastly, innate lymphoid cells (ILC) are crucial for regional immunity. They were first described for their role in the development of lymphoid tissues and generating inflammation at barrier surfaces after infection or tissue damage. More recently, however, a role in the transition from innate to adaptive immunity, and in chronic inflammation, has been elucidated [4]. Along these lines, recent advances suggest that components of the intestinal microbiota can alter gut homeostasis and promote chronic inflammation, thereby leading to intestinal tumor development [5].

Humoral mechanisms of innate immunity include complement proteins, cytokines, and other plasma proteins such as collectins, ficolins and pentraxins. The complement system is a branch of the immune system which, through a biochemical cascade involving multiple proteins, clears pathogens through cell lysis and phagocytosis. Complement is likely to have a dual role in cancer, as on the one hand it contributes to protection through direct complement activation or as part of complement-dependent cytotoxicity of tumor-directed therapeutic antibodies. Alternately, the generation of C5a in the tumor microenvironment can attract myeloid-derived suppressor cells (MDSCs) and induce the generation of reactive oxygen and nitrogen species through the C5a receptor, which impairs the tumor-directed effect of T cells [6]. There is evidence that both acute and chronic inflammation may promote genetic abnormalities and cancer progression, suggesting that modulation of the innate immune system has the potential to impact our approach to cancer immunotherapy.

### Dendritic Cells

DCs lie at the interface of innate and adaptive immune responses. First described by Steinman and Cohn in 1973, DCs can develop from either myeloid of lymphoid hematopoietic lineages, giving rise to a multitude of subtypes each with unique function. Adding to the diversity of subtypes is the localization of the particular DC types within different tissues in the body, as the milieu is critical for defining the ultimate function of the DC and their capacity to facilitate an anti-tumor immune response [7].

DCs start off in an immature state, acting as sentinels in their respective tissues. At this stage of maturation, DCs are very phagocytic, constantly sampling their environment through pinocytosis, micropinocytosis, and receptor-mediated uptake. Innate receptors, including TLRs and scavenging receptors, can facilitate maturation and activation of these DCs, and direct the nature of the immune response they trigger. For example, ligation of endosomal receptors TLR7 or TLR9 in human plasmacytoid DCs leads to secretion of interferon alpha (IFNα) and an anti-tumor T-helper type 1 (T_H_1) response. Similarly, ligation of TLR3, TLR4, TLR5, TLR8 and TLR11 on human myeloid DCs leads to a T_H_1 response, with the secretion of the heterodimeric IL-12p70. On the other hand, ligation of TLR1, TLR2 or TLR6 in these human myeloid DCs leads to a tolerogenic T-helper type II (T_H_2) response, with low IL-12p70 and high IL-10 secretion [8]. In the absence of maturation or inflammatory signals, antigen uptake and presentation results in a regulatory and tolerant response. On the other hand, antigen uptake that occurs in the presence of DAMPs/PAMPs leads to DC maturation and a decrease in phagocytic ability. These mature DCs traffic from the periphery to the lymph nodes, where they present the antigen to T-cells within the lymph node. In the right immunogenic setting, DCs increase their cell-surface expression of MHCI, major histocompatibility complex class II (MHCII), CD80, CD86, and secrete a number of immunogenic cytokines and chemokines, which in turn may activate T-cells and promote an anti-tumor T_H_1 response.

Given the ability of DCs to skew immune responses, there has been keen interest in these cells for the development of immunogenic antitumoral therapies. In 2010, the FDA approved a DC-based immunotherapy (sipuleucel-T) for men with metastatic castrate resistant prostate cancer. This cell-based vaccine uses ex-vivo pulsed autologous peripheral blood monocytes, incubated with a fusion protein that links Prostatic Acid Phosphatase (the target antigen) to GM-CSF (a factor that serves to mature monocytes towards DCs). In a randomized controlled trial in men with castrate resistant prostate cancer, sipuleucel-T improved survival by a median of 4.1 months compared to placebo (*p* = 0.03). This immunotherapy was also well tolerated and safe, with the main side effects limited to chills, fever, and headache [9].

Overall, DC-based therapies have led to suboptimal clinical responses, and to date, sipuleucel-T remains the only FDA approved DC-based immunotherapy. However, many unanswered questions remain when it comes to our fundamental understanding of this approach. For example, in the trials utilizing ex vivo expanded autologous DCs, (1) what DC subtype is the most potent, (2) what are the optimal culture and maturation conditions, (3) what is the best way to load tumor antigen, (4) what is the ideal dose, and route of administration? In contrast to ex vivo expanded autologous DCs, in vivo targeting of tumor antigens tethered to antibodies recognizing DC-specific receptors is another strategy being investigated.

### Adaptive immunity

Two groups of immune cells, B-cells and T-cells, work in concert to generate the adaptive immune response, which is characterized by three distinct features: diversity, specificity and memory. T-cells and B-cells each are capable of recognizing a diverse repertoire of specific antigens, but accomplish this in different ways [10]. T-cells, by means of the T-cell receptor (TCR), recognize peptide fragments (epitopes) presented on the surface of cells in the context of MHCI/MHCII surface receptors. B-cells recognize antigen through either B-cell receptor (BCR), or a soluble, secreted component of the BCR called an “antibody,” which may directly bind antigens found in serum [11].

Unlike most other receptors in the body, the DNA sequences of the TCR and BCR are not merely encoded by an individual’s germline DNA. Rather, the diversity and specificity of unique receptors are generated by a somatic recombination process. During this recombination process, Variable, Diversity, and Joining (VDJ) gene segments are randomly recombined, creating the genetic diversity of the TCR and BCR sequences. Following this recombination process, the estimated number of TCRs and BCRs that can be generated in an individual is of the order of approximately 2.5 × 10 [7]. Thus, the recombination of genes generates the underlying diversity of TCRs and BCRs, which then enables a diverse immune response against a broad repertoire of potential antigens [11].

These diverse cells, each with their own unique TCR or BCR, are present in the immune milieu, constantly surveying for the presence of their target “cognate” antigen. When a T-cell or B-cell binds to its cognate antigen under the right conditions, it will proliferate to create a clonal population, thus allowing for the generation of a potent, antigen-specific effector response. In addition to generating this effector population, cells with enhanced longevity are generated which provide “immunologic memory” and protection if the antigen is encountered again later.

B-cells generate what is termed a ‘humoral response.’ B-cells initially harbor BCRs on their surface, and are triggered to proliferate when they encounter a specific antigen. This process leads to the generation of plasma cells that generate large quantities of antibody, which are soluble BCRs that are released from the surface of plasma cells into the circulation. Antibodies have a basic Y-shaped structure, consisting of 2 heavy chains in the middle, flanked by two smaller light chains on the outside. The fork end of this structure forms the antigen binding site or “Fab” (fragment of antigen-binding) region. The other end of the antibody molecule consists of the two heavy chain regions alone, called the “Fc” region (constant fragment). The constant region defines the subtype or “isotype” of antibody, each with a distinct function. IgA antibodies are involved in the defense of mucosal surfaces. IgD antibodies are found mainly on naïve B-cells before they encounter antigen. IgE antibodies are generated in response to parasitic infections and allergic responses. IgG antibodies represent the host’s response to antigens encountered in the past, and account for post-vaccination antibody responses. Finally, IgM antibodies are a pentamer and are generated acutely during infection, preceding IgG. Antibodies may bring about their effect against pathogens through a number of different mechanisms, including neutralization (direct binding to pathogen), opsonization (binding of pathogen to facilitate phagocytosis), precipitation (binding pathogens into a formation), and complement activation [10].

T-cells are divided into several subtypes, defined by their function and expression of surface proteins. Cytotoxic T-cells express CD8, and upon activation can proliferate and directly kill infected or cancerous cells. Activation of cytotoxic T-cells requires the presence of two signals: the first signal (signal 1) consists of recognition of foreign antigen in the context of TCR and MHCI molecules, which are present on all human cells. Signal 2 is generated by a variety of co-stimulatory (for example CD28) and co-inhibitory molecules (for example cytotoxic T-lymphocyte antigen 4, CTLA4) that bind to ligand on target cells. An abundance of co-inhibitory signals may lead to a “tolerogenic” state by which cytotoxic T-cells lose their cytotoxic effector function, whereas co-stimulatory signals may promote cytotoxic effector function [12].

Helper T-cells express CD4 and facilitate a coordinated immune response involving cytotoxic T-cells, B-cells, and the innate immune arm. When naïve (antigen-inexperienced) CD4^+^ T-cells are stimulated with antigen in the context of MHCII molecules (found on antigen presenting cells such as B-cells, macrophages, and dendritic cells), the helper cell may differentiate into a T_H_1, T_H_2, T-helper type 17 (T_H_17), or regulatory (T_reg_) T-cell based upon the cytokine milieu [13]. T_H_1 cells secrete IFNγ and support antiviral and antitumor responses. T_H_2 cells secrete IL-4 and IL-13, support antiparasitic responses, and may hamper the anti-tumor response. The role of T_H_17 cells and other subtypes are being elucidated. Finally, T_regs_ inhibit and dampen an immune response, and have been attributed to tumor immune escape [13].

### Adoptive therapy

Adoptive cellular therapy is a field that involves ex vivo manipulation of autologous T cells and re-infusion in order to generate a robust anti-tumor response. Manipulation of T cells consists of either gene insertion of a chimeric antigen receptor (CAR) or engineered TCR, or the expansion of endogenous tumor infiltrating lymphocytes (TILs).

The primary components of a CAR are 1) an extracellular single-chain variable fragment (the “antigen-specific” side of an antibody) targeting a tumor antigen such as CD19 on lymphocytic leukemias; 2) a transmembrane linker domain; and 3) an intracellular CD3z (intracellular) domain, conferring downstream TCR signaling upon binding to tumor antigen. CAR-engineered T-cells are capable of binding tumor and being directly activated without the need for antigen-binding via the traditional TCR:MHC complex. Second-generation CARs have been enhanced by the addition of a co-stimulatory domain such as CD28 or 4-1BB. CAR-engineered T-cells are potent, however they may have short in vivo persistence.

The first published clinical example of CAR T-cell therapy in humans was a study of anti-CD19 CAR T-cells for the treatment of chronic lymphocytic leukemia (CLL), whereby 2 of 3 treated patients had durable remissions [14]. This study showed that CD19-directed CAR T-cells are capable of killing >1000 tumor cells per infused T-cell, and in at least some cases, there was long-term detection of CAR T-cells in blood by PCR, which correlated with durability of remission [15]. Subsequent enrollment on the CLL trial, however, proved that most patients did not experience dramatic long term remissions. This is in contrast to ALL, whereby infused anti-CD19 CAR T-cells demonstrated robust efficacy. The University of Pennsylvania trial of primarily pediatric ALL demonstrated a 90 % complete remission rate with a 6-month overall survival of 78 % [16].

TCR gene transfer has also been attempted as an alternative to CAR T-cell therapy. Endogenous TCRs specific to tumor-associated antigens are oftentimes low affinity and may not generate sufficient signal to mount a successful anti-tumor immune response. With gene transfer technologies, high-affinity TCR genes can be transferred to autologous T-cells, which then have the capacity to impart viral-like (strong) affinity against tumor-specific peptide:MHC complexes. In the clinic, this technique has been less successful than CAR therapy. In a study of TCR gene transfer against the cancer-testis antigen MAGE-A3, two patients died of cardiogenic shock. Autopsy studies demonstrated that the adoptive T-cells cross-reacted to a cardiomyocyte protein (titan), leading to death of cardiac myocytes. Similarly, in another study of anti-MAGE-A3 T-cells in 9 patients with various solid malignancies, 5 of 9 patients experienced objective response, however 3 patients developed severe neurologic adverse events, resulting in 2 treatment-related deaths. Brain biopsies demonstrated possible MAGE protein expression in the brain, once again highlighting the challenge of achieving tumor specificity with engineered TCRs [17–19].

Ex vivo expansion and re-infusion of TILs are the third type of adoptive T cell therapy. Great advances have recently been made in the culture process to allow for the less laborious and more rapid expansion of clinically relevant quantities of TILs. In Europe there is a phase III trial of TILs for melanoma with the hopes to generate robust efficacy data and gain approval of TILs as a standard therapy.

After many years, adoptive cellular therapy may be headed towards FDA approval based on the early stage clinical trials using CD19 targeted CAR T cells for ALL and non-Hodgkin lymphoma. Gene transfer of TCRs may also be headed towards later stage trials for sarcoma and melanoma. Future questions in the field include how to combine adoptive T cell therapy with other types of immunotherapy, how to scale up and streamline manufacturing, and the challenge of identifying novel targets with minimal “on target, off tumor” toxicity.

### Anti-tumor antibodies

Monoclonal antibodies (mAb) have been employed in a diversity of roles for the treatment of cancer. The “magic bullet concept” initially postulated the use of antibodies to target tumors [20]. Technical advances including the development of hybridoma technology and genetic approaches allowing the construction of “humanized” or fully human antibodies have facilitated the development of antibodies for use in the clinic.

The basic antibody structure consists of two heavy chains and two light chains, each of which has a variable portion and a constant region. The variable region of the heavy and light regions forms the antigen biding sites (Fab); each antibody has two Fab sites. The complementarity determining region (CDR) is within the antigen binding site and is critical for conferring specificity to the antibody. For example, rituximab and ofatumamab are both therapeutic mAb that target different sites of CD20 as they have different CDR regions [21]. Additionally, the class or subclass of the immunoglobulin can impact its function. Most antibodies developed for the clinic are in the IgG class, which is subdivided into IgG1, IgG2, IgG3, and IgG4 subclasses. IgG1 mAb subtypes typically have the most significant antibody-dependent cell-mediated cytotoxicity (ADCC, i.e. binding and killing of antibody-bound targets via NK cells and other effector cells), whereas mAbs of the human IgG4 subtype are thought typically have minimal ADCC, which may be consideration in the development of antibodies for clinical application.

Monoclonal antibodies for clinical use are classified and named based on the percentage of the murine component: murine (−omab), chimeric (−ximab), humanized (−zumab), and human (−umab). Human mAb are produced by using a phage antibody library, transgenic mice, or immortalized human memory B cells. Humanized and fully human mAb are preferred as they are less immunogenic.

Monoclonal antibodies act on their targets in an Fc dependent or an Fc independent fashion. Fc dependent mechanisms of action are as follows: ADCC, antibody-dependent cellular phagocytosis (ADCP), and complement dependent cytotoxicity (CDC). On the other hand, direct apoptosis, agonistic and antagonistic mediated interactions are Fc independent. Various strategies have been employed to enhance the efficacy of mAb such as glyco-engineering of the Fc portion in order to enhance ADCC, for instance obinutuzumab (GA101) as compared to rituximab [22]. Additionally, therapeutic antibodies can be enhanced by conjugating them to radioisotopes, cytotoxic molecules, or cytokines, thereby improving the likelihood of cell kill. One example of an antibody-drug conjugate (ADC) is trastuzumab emtansine (T-DM1), which is an antibody targeting anti-human epidermal growth factor receptor 2 (HER2), conjugated to the cytotoxic molecule, DM1. T-DM1 improves survival and is FDA-approved for HER2-overexpressing metastatic breast cancer, and is being additionally evaluated in both early stage breast cancer and HER2-overexpressing gastric cancer [23].

In addition to conjugating antibodies to cytotoxic agents, bispecific antibodies have been developed which link the variable regions of two antibodies together, thereby creating a construct specific to two antigens. The FDA-approved drug, blinatumomab, is specific to both CD3 and CD19, and functions to engage T-cells with malignant CD19-positive leukemia clones, directly leading to T-cell activation and cytolysis of the adjacent leukemia cell [24].

### Obstacles to immunotherapy

The concept of immune surveillance was described in the 1950s by L. Thomas and M. Burnet. They theorized that T cells played a pivotal sentinel role in the immune system’s response against cancer, potentially recognizing and eliminating cancerous cells. Later, Schreiber and colleagues proposed a hypothesis called the three E’s of cancer immunoediting: Elimination, Equilibrium, and Escape [25]. These three E’s illustrate how, with selective immune pressure, cancers can be sculpted over time to become progressively resistant to the immune response. During the elimination phase, adaptive and innate immune arms work in tandem to recognize and destroy tumors. However, during the equilibrium phase, immune pressure results in the acquisition of resistance mechanisms by tumors that allow for survival of cancer in a steady state. Finally, over time, during the escape phase, tumor variants may emerge that are no longer recognized by adaptive or innate immune arms, allowing for outgrowth of tumors and clinical manifestation of cancer.

The methods by which tumors evade immune elimination are an active area of research. These can be categorized into tumor-intrinsic and tumor-extrinsic. Tumor-intrinsic mechanisms can include antigen loss, MHC loss, secretion of immunosuppressive cytokines or expression of cell-surface markers such as programmed death ligand 1 (PD-L1) that may alter T cell function. Tumor-extrinsic factors involve geographic barriers as well as a range of suppressive or regulatory immune cells including regulatory T cells and a heterogenous population of MDSCs and alternative activated M2-like tumor-associated macrophages (TAMs). T_regs_ are characterized by expression of the transcription factor FOXP3 and are critical for the prevention of autoimmunity and the maintenance of immune homeostasis. T_regs_ modulate the immune response by a variety of mechanisms. Potential opportunities for targeting T_regs_ include T_reg_ depletion via blocking antibodies or anti-CD25 immunotoxin, modification of trafficking or exploitation of T-cell plasticity [26]. Recent data suggests that CTLA-4 blocking antibodies such as ipilimumab may act, in part, via their role in depleting T_regs_ from the tumor microenvironment [27].

MDSCs are a population of immune derived cells that inhibit T cell and dendritic cell function via a variety of mechanisms [28]. These cells may play an important roll in immune editing in metastatic cancer. For example, metastatic melanoma patients have been observed to have increased quantities of MDSCs [29]. These CD14+ MDSC were also shown to directly suppress T-cell proliferation ex-vivo. In addition, MDSC are associated with poorer survival outcomes after ipilimumab. TAMs are monocytes that are recruited to the tumor microenvironment. Cytokines secreted by the tumor can polarize recruited monocytes to either resemble M1 macrophages which have tumoricidal activity or, more commonly, to resemble M2 macrophages. These M2-like TAMs can shape the tumor microenvironment by secreting a variety of cytokines for tissue remodeling, enhanced invasion and metastasis and increase immune suppression (mostly via IL-10 production) [30]. There are a number of compounds that are being explored to target MDSCs and TAMs, for example colony-stimulating factor 1 receptor (CSF-1R) blocking agents that are currently under development, however, specificity for these agents remains a significant challenge.

Lastly, one of the now well-known regulatory mechanisms which serve to dampen or shut down T-cell responses are immune checkpoint molecules expressed on the T-cell surface, including CTLA-4 and programmed death 1 (PD-1). Two anti-PD-1 antibodies, pembrolizumab and nivolumab, and one anti-CTLA4 antibody, ipilimumab, are FDA approved for the treatment of metastatic melanoma. There continues to be active investigation into new blocking antibodies of inhibitory receptors such as lymphocyte activation gene 3 (LAG-3).

### Cancer vaccines

Much like vaccines for the prevention of infectious disease, vaccines for cancer are designed to induce an adaptive immune response to an administered antigen. A key difference, however, is that cancer vaccines have generally been studied as therapeutic, rather than prophylactic, agents. In addition, unlike pathogens, for which non-self antigens may be relatively easily identified and defined, the identification of appropriate antigen targets to generate an effective anti-tumor response is more challenging. Tumor antigens may be categorized as tumor associate antigens (TAA) which are present in tumor cells, but may also be present in some normal cells. An example of a TAA would be MAGE, found in many melanomas, but also present in the testis. Alternatively, tumor antigens may be categorized as tumor specific antigens (TSA), antigens that are completely unique to the tumor and not found in normal tissue. Antigens generated by random mutations in the tumor cells would be an example of TSAs. The quantity and quality of TSA and TAA antigens varies widely among cancers.

While cancer vaccines have been investigated for decades, the first approved treatment for human cancer based on the principle of vaccination is sipuleucel T (FDA-approved in 2010). This approach, which is now an accepted therapy for patients with advanced prostate cancer, begins with the harvesting of a patient’s antigen presenting cells, which are then exposed to tumor antigen ex vivo, and subsequently returned to the patient. Despite the experience with sipuleucel-T, the more conventional approach of administering an antigen into the patient in order to trigger a productive adaptive immune response is ongoing. A critical component of such vaccination is the so-called “adjuvant”, a second agent that is co-administered to enhance the response to the antigen. There are multiple types of adjuvants, including those designed to prolong antigen release, to protect the antigen from degradation, to increase antigen uptake by antigen presenting cells, or to induce an inflammatory microenvironment.

Despite much research and clinical activity in this area, so far most cancer patients do not achieve tumor regression with vaccination, even when tumor-specific T-cells are induced. Some possible explanations for this are that 1) too few antigen-specific T-cells develop; 2) the selection of which antigens to target is sub-optimal; 3) the vaccine induced T cells have sub-optimal anti-tumor activity 4) T-cells are being suppressed by tumor-derived immunosuppressive signals; 5) or activated T-cells are prevented from entering the tumor [31]. Fascinatingly, it has also been demonstrated that vaccines may blunt their own activity by diverting antigen-specific T-cells away from tumors to sites of vaccination where they undergo apoptosis. Fortunately, this obstacle may be overcome by using dissipating vaccine formulations [32].

A history of extensive experience with cancer vaccines, and the advent of immune checkpoint inhibitors with potent anti-tumor activity, has raised hopes of successfully combining vaccination with other immunotherapies. While not all such combinations are likely to be effective, there are many studies showing that this strategy can indeed boost vaccine potency.

### Immune checkpoint antibodies

Immune checkpoint modulation has transformed the treatment of cancer, with the FDA-approval of the CTLA-4 blocking antibody ipilimumab and the PD-1 blocking antibodies nivolumab and pembrolizumab. CTLA4, the prototypical co-inhibitory checkpoint receptor, attenuates the T-cell response by inducing inhibitory downstream signaling and competitively binding with the B7 ligand on antigen presenting cells [33]. Blockade of CTLA4 improves survival in both mice [34] and in humans: in a phase III trial, melanoma patients with previously treated, advanced melanoma received ipilimumab 3 mg/kg with or without glycoprotein (gp) 100 peptide vaccine vs gp100 peptide vaccine alone [35]. Median overall survival (OS) in the ipilimumab and ipilimumab + gp100 cohorts was 10.1 and 10.0 months, respectively, versus 6.4 months for the gp100 control arm (*p* < .001). Following ipilimumab, survival appears to be durable, as indicated by a plateau in the survival curve beginning around year 3 (21 % 3-year survival) and persisting for >10 years [36]. Immune-related toxicities such as colitis, hepatitis, pneumonitis, or hypophysitis may be serious, but can be mitigated with careful monitoring of symptoms and algorithm-guided early intervention, for example with corticosteroids [37].

PD-1 is another immune inhibitory receptor expressed on T-cells with therapeutic potential [38]. The anti-PD-1 antibodies nivolumab and pembrolizumab have activity in patients with both ipilimumab-naïve and ipilimumab-treated advanced melanoma and have been recently FDA approved in the latter patient population [39,40]. In addition, PD-1 or PD-L1 blocking antibodies have demonstrated activity in a wide variety of additional tumor types including non-small cell lung cancer (NSCLC), renal cell cancer, bladder cancer, and Hodgkin’s lymphoma among others. Nivolumab has recently been approved by the FDA for the treatment of NSCLC of squamous histology based upon the results of a Phase 3 study showing a benefit in overall survival of 3.2 months for patients who received nivolumab (clinical trial NCT01642004).

The combination blockade of CTLA4 and PD-1 pathways may be more efficacious in promoting antitumor immunity than either agent alone [41]. In a phase I study of simultaneous administration of nivolumab and ipilimumab in patients with advanced melanoma, dramatic tumor reduction (>80 % tumor volume) was observed in 42 % of the initial 53 patients with advanced melanoma, with a 2-year survival rate of 88 % in the optimal dosing cohort of nivolumab, 1 mg/kg, and ipilimumab, 3 mg/kg [42,43]. These checkpoint inhibitors are also being evaluated in a variety of tumor types including lung cancers, urologic malignancies, hematological malignancies.

### Monitoring T-cell and B-cell responses to immunotherapy

Laboratory assays may be used to monitor B-cell and T-cell function in response to immunotherapy, and may one day serve as biomarkers to inform clinical decision-making. The traditional method of monitoring B-cell function is by measuring serum antibody reactivity by enzyme linked immunosorbent assay (ELISA). By this method, antibody production against cancer-associated antigens have been detected and monitored in cancer patients. For example, sero-reactivity against NY-ESO-1, a cancer-testis antigen expressed by some melanomas—has been associated with clinical benefit following anti-CTLA4 therapy [44]. One limitation of ELISA, however, is that *a priori* knowledge of the antigenic target is necessary. Alternatively, next generation protein microarrays provide the opportunity to simultaneously measure sero-reactivity against thousands of antigens, allowing for possible antigen discovery. These microarrays have high concordance with ELSA, and have been used to identify antigenic targets and sero-reactivity patterns in ovarian cancer, pancreatic cancer, and other cancers [45]. Alternatively, an emerging method to monitor B-cell function is to enumerate ectopic tumor-associated lymphoid structures (TLS) found within tumors. TLS contain the hallmark components of the lymphoid follicle (i.e. naïve B-cells, germinal center B-cells, and somatic hypermutation/class switch machinery), have been associated with favorable prognosis, and contain B-cells that secrete auto-antibodies against known tumor-associated antigens such as NY-ESO-1 [46,47].

Multiple methods have been developed to monitor T-cell function, for example polychromatic flow cytometry to quantify the degree of peripheral blood T-cell activation as measured by expression of ICOS [48]. However, assessment of TILs may be additionally informative. Quantification of CD3 (T-cells), CD8 (cytotoxic T-cells) and CD45RO (memory T-cells) by immunohistochemistry is highly prognostic of recurrence across multiple tumor types [49]. For example, early stage colorectal tumors with dense CD8+ and CD45RO+ infiltrates have dramatically lower recurrence (4.8 % 5-years recurrence) compared to tumors with low infiltrates (75 % 5-years recurrence) [50]. These parameters have formed the basis of the proposed “immunoscore,” which if validated may inform the clinical decision to treat a patient with adjuvant therapy. More sophisticated analytic tools of TILs are being developed and may provide additional prognostic information or enhanced understanding of the interplay of various immune subsets. For example, RNA transcriptome analysis has been used to characterize the transcriptional profiles of various immune cellular subsets. By RNA-seq, T_H_17 transcriptional profiles were associated with adverse prognosis in colorectal cancer. Furthermore, tumors of different T-stages each exhibited a distinct immunologic transcriptional profile, suggesting temporal evolution of interplay between the tumor and immune system [51].

Conventional immune monitoring platforms measure only the average signal across all the cells within a sample. However, next-generation techniques may allow for characterization of individual immune cells. Advanced flow cytometry techniques allow for sorting and classification of cells by upwards of 20 different parameters. Alternatively, mass cytometry utilizes antibody stains conjugated to rare-earth metal isotopes that emit non-overlapping signals, thus increasing the theoretical limit of characterization to upwards of 100 distant parameters [52]. Finally, “microfluidics” platforms and other technologies are being developed to isolate and perform whole genome and RNA sequencing on individual cells. These tools, while in their infancy, will provide a wealth of mechanistic information, and may one day be used to predict response to immunotherapy and guide clinical decision-making [53].

### Immunotherapy clinical trial design

The rapid expansion of the immuno-oncology field has likewise led to the identification of an ever-expanding list of critical issues that require further investigation, such as identification of the molecular mechanisms of antitumor efficacy and off-target toxicity, identification of predictive/prognostic biomarkers, and the identification of novel therapeutic targets. One approach to investigating these unanswered questions is designing immunotherapy trials that integrate surgical resection or tissue sampling during the course of treatment. This allows for an iterative process of hypothesis generation from patient samples that can be then tested in a more tightly controlled laboratory setting and lead to novel future trials.

An illustrative example of this approach is the series of studies that identified ICOS as a pharmacodynamic marker of CTLA4 inhibition [48] and a potential therapeutic target [54]. Initially, twelve patients with bladder cancer were treated with 2 cycles of ipilimumab in the neoadjuvant setting [55]. Increased levels of ICOS expression on CD4+ cells (CD4 + ICOS^hi^) in both tumor tissue and plasma were seen, and sustained increases of circulating CD4 + ICOS^hi^ T cells were associated with clinical benefit [55]. Increased frequency of CD4+ICOS^hi^ T cells was 71% sensitive and 96% specific as a pharmacodynamic biomarker of anti-CTLA4 treatment across cancer subtypes in this small initial cohort [48], but larger studies in over 200 patients have demonstrated ICOS+ CD4 T cells to have sensitivity of 91% and specificity of 96% as a pharmacodynamic biomarker of anti-CTLA-4 therapy (unpublished data, PS).

Mouse models were then engineered that suggested ICOS is necessary for optimal anti-tumor efficacy of CTLA4 treatment [56]. ICOS signals through the PI3K pathway to increase Tbet expression, which underlies the development of T_H_1 cells that lead to anti-tumor responses [57]. Ongoing preclinical work with an ICOS-ligand expressing vaccine in murine models shows improvements in anti-tumor responses to CTLA4 inhibition [54]. Together, these data suggest that one strategy to improve clinical outcomes with CTLA4 blockade may be to increase ICOS expression and signaling.

As the development of ICOS shows, varied clinical trial designs can be used to investigate biomarkers and mechanisms of tumor response or resistance that will lead to the next phase of clinical trial design. As the field of immuno-oncology continues to expand, these biomarker-driven studies will serve as invaluable tools for discovery and design of new therapies.

## Conclusion

As foreshadowed by the work of Drs. William B. Coley and Lloyd J. Old, advances in our understanding of the innate and adaptive immune response have finally translated into remarkable gains in clinical benefit for patients across of variety of malignancies. These gains are occurring across several classes of immunotherapy: for example, the FDA has approved therapeutic immune checkpoint antibodies for metastatic melanoma and lung cancer, cellular vaccines for prostate cancer, and bispecific antibodies for leukemia. Additional therapies such as CAR-engineered T-cells for leukemia are likely soon to follow. Looking forward, clinical advances are likely to accelerate as a result of broadening our application of these therapeutic strategies to different tumor targets (for example employing CAR-engineered T-cells for solid tumors, or checkpoint antibodies for leukemias), or combinatorial immunotherapy approaches (for example enhancing CAR-engineered T-cells by co-administering immune checkpoint antibodies). Future challenges will be to understand the optimal sequencing of numerous effective immune therapies and alternative treatment approaches, improving access to immune therapy, and developing bioinformatics approaches to translate next-generation immune monitoring data into clinical use [58,59].

## Abbreviations

ADC: Antibody drug conjugate
ADCC: Antibody-dependent cellular cytotoxicity
ADCP: Antibody-dependent cellular phagocytosis
ALL: Acute lymphocytic leukemia
BCR: B-cell receptor
CAR: Chimeric antigen receptor
CDC: Complement dependent cytotoxicity
CDR: Complementarity determining region
CLL: Chronic lymphocytic leukemia
CTLA4: Cytotoxic T-lymphocyte antigen 4
DAMP: Damage-associated molecular pattern
DC: Dendritic cell
ELISA: Enzyme linked immunosorbent assay
Fab: Antigen binding fragment
Fc: Constant fragment
FDA: Food and drug administration
Gp: Glycoprotein
HER2: Human epidermal growth factor receptor
ICOS: Inducible co-simulator
IFN: Interferon
IL: Interleukin
LAG3: Lymphocyte activation gene 3
mAb: Monoclonal antibody
MDSC: Myeloid derived suppressor cell
MHCI: Major histocompatibility complex class I
MHCII: Major histocompatibility complex class II
NHL: Non-Hodgkin lymphoma
NK: Natural killer cell
PAMP: Pathogen-associated molecular pattern
PD-L1: Programmed death ligand 1
PRR: Pattern recognition receptor
SITC: Society of the immunotherapy of cancer
TAA: Tumor associated antigen
TAM: Tumor associated macrophage
TCR: T-cell receptor
T-DM1: Trastuzumab emtansine
T_H_1: T-helper Type 1 cell
T_H_2: T-helper Type 2 cell
TIL: Tumor infiltrating lymphocyte
TLR: Toll-like receptor
TLS: Tertiary lymphoid structure
VDJ: Variability, Diversity, and Joining
